# Subcortical volumes, frontal cortical thickness, and pro-inflammatory cytokines in schizophrenia versus methamphetamine-induced psychosis

**DOI:** 10.1007/s11682-025-01022-9

**Published:** 2025-05-28

**Authors:** Lauren Blake, Kimberley C. Williams, Anne A. Uhlmann, Henk Temmingh, Antoinette Burger, Dan J. Stein, Petrus J. W. Naudé

**Affiliations:** 1 Department of Psychiatry and Mental Health, Faculty of Health Sciences, University of Cape Town, Main Road, Observatory Cape Town, Cape Town, 7935 South Africa; 2 Department of Child and Adolescent Psychiatry and Psychotherapy, Faculty of Medicine, Technische Universität Dresden, Dresden, Germany; 3 Neuroscience Institute, University of Cape Town, Cape Town, South Africa; 4 South African Medical Research Council Unit on Risk and Resilience in Mental Disorders, University of Cape Town, Cape Town, South Africa; 5 University of Missouri, Columbia, United States of America

**Keywords:** Subcortical volumes, Cortical thickness, Psychotic disorders, Neuroinflammation

## Abstract

Schizophrenia is associated with alterations in subcortical volumes, cortical thickness and pro-inflammatory cytokines, that may correlate with clinical features. However, analogous work on methamphetamine-induced psychosis is lacking. This study examines subcortical volumes, frontal cortical thickness and pro-inflammatory cytokines in schizophrenia and methamphetamine-induced psychosis.

Diagnosis and symptom severity were determined using the Structured Clinical Interview for Axis I Disorders and the Positive and Negative Syndrome Scale, respectively. Structural T1-weighted images were acquired using a 3-Tesla magnetic resonance imaging scanner. Serum peripheral cytokine concentrations were measured using a multiplex bead array.

Schizophrenia (*n* = 36) and methamphetamine-induced psychosis (*n* = 27) participants showed decreased left amygdala volumes and frontal cortical thickness compared to healthy controls (*n* = 32). Schizophrenia participants had increased bilateral caudate, putamen, and nucleus accumbens volumes compared to controls, and greater right globus pallidus and nucleus accumbens volumes compared to the methamphetamine-induced psychosis group. No significant differences were found in cytokine levels between groups or associations with neuroimaging measures.

The novel discovery of increased globus pallidus and nucleus accumbens volumes in schizophrenia group compared with methamphetamine-induced psychosis group may show important distinctions in the neurobiology between these conditions. Future investigations should employ larger sample sizes, incorporate longitudinal study designs, and integrate magnetic resonance spectroscopy which may show important neurometabolic signatures in these brain regions in methamphetamine-induced psychosis.

**Supplementary Information:**

The online version contains supplementary material available at 10.1007/s11682-025-01022-9.

## Introduction

Methamphetamine-induced psychosis (MAP) presents with clinical symptomatology which is indistinguishable from that of schizophrenia including, positive and negative symptoms of psychosis, with no difference in the severity of symptoms (Medhus, 2014). According to the Diagnostic and Statistical Manual of Mental Disorders-IV (DSM-IV) states that if a methamphetamine user has persistent psychotic symptoms after being abstinent from methamphetamine for 6 months, the diagnosis is effectively changed from substance-induced psychosis to schizophrenia (American Psychiatric Association. 2013). These clinical overlaps strongly suggest shared neurobiological mechanisms, positioning MAP as a potentially valuable model for studying the pathophysiology of schizophrenia. However, despite these parallels, few studies have systematically investigated the shared and distinct biosignatures of these conditions, highlighting a critical gap in our understanding of their neurobiological convergence and divergence.

Structural magnetic resonance imaging (MRI) studies have shown differences in subcortical gray matter volumes, in schizophrenia, which have been associated with clinical symptom profiles (Duan et al., 2021; Fan et al., 2019; Koshiyama et al., 2018; Makowski et al., 2017; Rajarethinam et al., 2001). In schizophrenia, decreased hippocampal volume has been associated with negative symptoms and hallucinations (Duan et al., 2021; Rajarethinam et al., 2001), with decreased amygdala-hippocampal complex associated with negative symptoms of psychosis (Rajarethinam et al., 2001). Furthermore, increased basal ganglia volumes have been consistently observed in schizophrenia compared to healthy controls (Chakos et al., 1994; Corson et al., 1999b; Lauer et al., 2001). Alterations in basal ganglia volumes have also been associated with clinical symptom profiles (Duan et al., 2021; Fan et al., 2019; Koshiyama et al., 2018; Makowski et al., 2017; Rajarethinam et al., 2001) including positive and negative symptoms (Li et al., 2018). Previous studies have reported increased basal ganglia volumes in schizophrenia patients, which have been attributed to antipsychotic treatment (Emsley et al., 2023). Increased basal ganglia volumes in schizophrenia have also been attributed to typical antipsychotics, including increased putamen and caudate volumes (Chakos et al., 1994; Jørgensen et al., 2016). In support of these findings, antipsychotic-naïve schizophrenia individuals (Corson et al., 1999a; Keshavan et al., 1998) and individuals treated with atypical antipsychotics (Lang et al., 2004) display decreased basal ganglia volumes. Differences in cortical thickness of the frontal and temporal lobes have also been observed in individuals with schizophrenia (Kuperberg et al., 2003; Nenadic et al., 2015; Rimol et al., 2010, 2012; Sugihara et al., 2017; van Erp et al., 2018), with the frontal cortex being most affected (van Erp et al., 2018). While structural neuroimaging studies of MAP are limited, there is some evidence of increased cortical thickness in MAP compared to individuals with methamphetamine dependence (Uhlmann et al., 2016) and decreased hippocampal and amygdala volumes in MAP compared to healthy controls (Orikabe et al., 2011). However, no studies have directly compared subcortical volumes and cortical thickness in schizophrenia to that in MAP.

Schizophrenia is further characterized by a number of alterations in immune biomarkers. Alterations of peripheral blood inflammatory cytokines (e.g. TNF)-α, IL-1β, IL-6, IL-8, IL-10, IL-12, and IFN-γ) have been associated with clinical outcomes in schizophrenia (Kose et al., 2021; Pillinger et al., 2019). However, few studies have investigated immune markers in MAP, and none have directly compared immune markers in schizophrenia to those in MAP. Given findings of associations between neuroimaging measures and cytokine levels in schizophrenia (Wu et al., 2019), and evidence of neuro-inflammation in laboratory models of MA use (Davidson et al., 2022; Shi et al., 2022), investigation on associations between neuroimaging measures and cytokines in MAP can advance our understanding on the potential functions of neuro-inflammation in schizophrenia and MAP.

This study aims to compare subcortical volumes, cortical thickness, and cytokine levels in individuals with schizophrenia, MAP, and healthy controls. We also assessed associations of neuroimaging measures with illness duration, symptom severity, and pro-inflammatory cytokines in schizophrenia and MAP, and with duration of methamphetamine use in MAP.

We hypothesize that there will be decreased hippocampal and amygdala volumes in both schizophrenia and MAP, based on previous literature (Duan et al., 2021; Orikabe et al., 2011; Rajarethinam et al., 2001; Uhlmann et al., 2016) and that these will be associated with increased illness severity in both schizophrenia and MAP groups and increased duration of methamphetamine use in MAP. In addition, we hypothesize that there will be increased basal ganglia volumes in schizophrenia, due to typical antipsychotic exposure, which has previously been reported (Chakos et al., 1994; Jørgensen et al., 2016) compared to MAP (Farnia et al., 2019) and healthy controls, and that these volumes will be associated with increased duration of illness. Thirdly, we hypothesize that there will be decreased frontal cortical thickness in both schizophrenia and MAP groups, based on previously literature (Uhlmann et al., 2016; van Erp et al., 2018; Walton et al., 2018) compared to healthy controls and that these will be associated with increased duration of illness in schizophrenia and MAP groups and with increased duration of methamphetamine use in the MAP group.

## Materials and methods

### Participants

In this exploratory study, participants between the ages of 20–40 years, were recruited from Valkenberg Hospital and Groote Schuur Hospital in Cape Town, and included 36 (8 females: 28 males) participants with schizophrenia, 27 (6 females: 21 males) participants with MAP and 32 (13 females: 19 males) healthy controls, matched for age and sex, were recruited from the general population in Cape Town.

Individuals with schizophrenia and MAP had clinical diagnosis confirmed with the Structured Clinical Interview for DSM-IV (SCID-I) (American Psychiatric Association. 2013), and symptom severity measured with the Positive and Negative Syndrome Scale (PANSS) (Kay et al., 1987). The SCID-I was also given to healthy controls to include only those without Axis-I disorders. Participants recruited for the MAP group were included if they had persistent presentation of psychosis due to methamphetamine use in which psychosis persisted beyond the initial period of intoxication or withdrawal (Camfield et al., 2024). Participants were excluded if they had a history of (1) chronic medical illness that required medical care or prescription medication, including severe brain trauma and intellectual disability; (2) were recently or currently pregnant or lactating; (3) were unable to undergo MRI because of metal implants, claustrophobia, or other reasons; (4) substance dependence other than nicotine dependence.

Written consent was obtained from all participants included in the study. The work adhered to human research guidelines as stipulated in the Declaration of Helsinki (World Medical, 2013). The protocol underwent ethical review and approval with the University of Cape Town’s Human Research Ethics Committee (UCT HSF HREC: 413/2016).

### Imaging procedures

Structural magnetic resonance imaging (sMRI) was conducted in the morning on the second day of the study at the Cape Universities Body Imaging Centre (CUBIC), using a 3T Siemens Skyra MRI scanner (Siemens Medical Systems, Erlangen, Germany). A 32-channel head coil was used. A high resolution, T1-weighted scans were acquired with a multi-echo MPRAGE structural scan with the following scan parameters: TR = 2530ms; graded TE = 1.69, 3.55, 5.41 and 7.27ms; flip angle = 7°; field of view (FOV) = 256 mm; voxel size = 1 × 1 × 1 mm; 6:39 min. Integrated Parallel Acquisition Techniques (iPAT) were used to obtain MRI scans using Generalized Autocalibrating Partially Parallel Acquisition (GRAPPA), with an acceleration factor PE of 2, reference lines PE of 32, acceleration factor 3D of 1 and reference scan mode of 1. The images were scanned in the sagittal plane producing 160 slices with a thickness of 1 mm x 1 mm x 1 mm.

### Image processing

Processing of all MRIs was performed using FreeSurfer v6.0 (Fischl, 2012; Incorporated, 1999) (http://surfer.nmr.mgh.harvard.edu/*)* using the Desikan-Killiany atlas, for cortical reconstruction and volume segmentation (Desikan et al., 2006). The processing pipeline included motion correction, skull-stripping, talairach transformation, segmentation of subcortical white matter and deep grey matter volumetric structures, intensity normalization, tessellation of the grey matter/white matter boundary, automated topology correction, and surface deformation and registration to the Desikan-Killiany atlas (Desikan et al., 2006). Reconstruction of the grey and white matter boundary and pial surface was performed to extract cortical thickness measurements (Fischl & Dale, 2000; Fischl et al., 2001; Segonne et al., 2007). Data and statistical summaries were generated using ENIGMA protocols (http://enigma.ini.usc.edu/protocols/imaging-protocols*)* and RStudio v 1.2.5054 (RStudio Incorporated, 2009–2020).

### Inflammatory markers

Blood samples were collected via venepuncture into serum tubes in the morning of the first day of the study. The tubes were kept at room temperature at for approximately 30 min to allow clotting and were subsequently centrifuged at 1,800 × g for 10 min. The separated serum was then aliquoted into cryovials and stored at -80 °C until analyses. The Luminex Milliplex^®^ MAP human cytokine / chemokine magnetic bead panel (HSTCMAG28SK07; Merck) was used to detect cytokines; TNF- IL-1 IL-6, IL-8, IL-10, IL-12p, and IFN-. The intra-assay coefficients of variation for each cytokine were all below 14%. All plates were analysed on the same day. Serum measures for IL-6 were below the detectable limit and were consequently excluded from statistical analysis.

### Statistical analyses

A priori power analysis, to determine the minimum required sample size (n) required, was conducted with G*Power 3 (Faul et al., 2007) as a function of the required power level (1 – β error probability = 0.8), the pre-specified significance level (α = 0.05), and the population effect size (d = 0.5) with an allocation ratio of N2/N1 = 1. The a priori power analysis yielded a required total sample size of *n* = 102, with statistical power of 0.81.

To compare brain measures across schizophrenia (*n* = 36), MAP (*n* = 27), and healthy controls (*n* = 32), we employed a multiple analysis of covariance (MANCOVA) for all brain regions, with intracranial volume (ICV) (Mathalon et al., 1993; O’Brien et al., 2011; Sanfilipo et al., 2004), sex, and age (Barnes et al., 2010; Crowley et al., 2018; Smith et al., 2007) as covariates for subcortical volume measurements. Age, sex, and intracranial volume (ICV) are known to influence brain structure, with males typically exhibiting larger ICVs and females showing greater gray matter volumes (Buckner et al., 2004; Courchesne et al., 2000; Ge et al., 2002; Nordenskjöld et al., 2014; Smith et al., 2007) In subcortical volume analysis, age, sex, and ICV were treated as independent variables, with ICV assumed to correlate linearly with each region of interest (ROI). As a stable marker of maximum brain volume post-neurodevelopment, ICV normalization helps clarify whether morphological variations in ROIs are due to head size or pathological changes (Caspi et al., 2020). However, cortical thickness is not dependent on ICV, with the development of the cortical thickness genetically independent from the development of cerebral cortical volume (Panizzon et al., 2009; Winkler et al., 2009). For this reason, only age and sex were used as covariates for frontal cortical thickness measurements. Education was not used as a covariate due to the complex relationship between educational attainment and brain structure, suggesting that variations in brain morphology can influence educational outcomes, while educational experiences may also shape brain development (Seyedsalehi et al., 2023) and that educational attainment, cognitive performance and cerebral cortical morphology are impacted by genetic variations (Ge et al., 2019). Furthermore, since both education attainment and socioeconomic status are correlated with cognitive function (Lee et al., 2003) as well as illness severity (Malliaris & Bonotis, 2021; Parrott & Lewine, 2005), including these as covariates could lead to overcorrection and bias in the interpretation of group differences. Adjusting for education as a covariate may remove the true relationship between brain structure and other variables in this study. Furthermore, education is more directly linked to functional plasticity and cognitive reserve than to brain structure itself. Adjusting for both education and socioeconomic status may have the potential to remove variance that is inherent to the psychotic disorders themselves (Miller and Chapman., 2001). Thereafter, a multiple analysis of variance (MANOVA) for parametric data and Kruskal Wallis analysis of variance (ANOVA) for non-parametric data to determine whether there were group differences. MANOVA was followed by Bonferroni post-hoc tests to determine which groups differed from each other. Effect sizes for group comparisons were calculated using partial eta squared for all parametric ANOVAs and eta squared for Kruskal Wallis ANOVAs at a confidence interval of 90% (Jané et al., 2024). Spearman’s Rank correlations (for non-normally distributed data) and Pearson’s correlations (normally distributed data) were performed within each group to determine associations of neuroimaging measures with symptom severity, illness duration, duration of methamphetamine use and cytokine concentrations. Significance levels were set at Rho of ± 0.5 and *p* < 0.01 (where the critical value for *r* = 0.449 when *p* = 0.01) to reduce Type I Error and increase stringency and strength of the associations between variables in addition to detecting moderate correlations as meaningful, which may be overlooked with a higher stringency Rho value of ± 1. The alpha value was then adjusted to $\:\alpha\:$ = 0.05 to balance the trade-off between Type I and Type II errors, this adjustment ensured a reasonable sensitivity for detecting meaningful effects and reducing the risk of false positives while maintaining rigorous statistical standards. This trade off ensured that only highly reliable correlations were reported in this study.

In a sensitivity analysis to determine whether there were differences between the schizophrenia participants who reported methamphetamine (*n* = 14) use to those that did not report methamphetamine use (*n* = 22), we employed t-tests for parametric brain regions and Mann Whitney U tests for non-parametric brain regions (Supplementary Tables 1, 2 and 3). Effect sizes were calculated using Cohen’s D for parametric data at a 95% confidence interval and biserial correlations *r* at *p* < 0.05.

A second sensitivity analysis was done to determine whether there were brain volume and cortical thickness differences between the schizophrenia participants treated with typical antipsychotics (*n* = 5) versus those treated with atypical antipsychotics (*n* = 21) and between MAP participants treated with typical antipsychotics (*n* = 6) versus those treated with atypical antipsychotics (*n* = 11). We employed t-tests for parametric brain regions and Mann Whitney U tests for non-parametric brain regions (Supplementary Tables 4–9). Effect sizes were calculated using Cohen’s D for parametric data at a 95% confidence interval and biserial correlations *r* at *p* < 0.05.

## Results

### Demographic and clinical variables

Table 1 shows that there were no significant group differences for age and sex, lower levels of school education were found in schizophrenia (H_(2,94)_ = 19.827, *p* = 0.002) and in MAP (H_(2,94)_ = 19.827, *p* = 0.00046), compared to healthy controls. Duration of illness was significantly longer in schizophrenia compared to MAP (*z* = 3.271, *p* = 0.0008) and the number of psychotic episodes were greater in schizophrenia than MAP (*p* = 0.038). Significant differences were found for the PANSS positive (H_(2,92)_ = 40.21, *p* < 0.0001), negative (H_(2,92)_ = 43.86, *p* < 0.0001), general (H_(2,92)_ = 32.99, *p* < 0.0001) and total scores (H_(2,92)_ = 46.64, *p* < 0.0001), with significant differences found between controls and schizophrenia (*p* < 0.0001) and, controls and MAP (*p* 0.00012).

**Table 1:** Demographics, duration of illness, clinical scales and methamphetamine use variables for control, schizophrenia and methamphetamine-induced psychotic groups

	CON (*n* = 32) 13 females: 19 males			SCHIZOPHRENIA (*n* = 36) 8 females: 28 males			MAP (*n* = 27) 6 females: 21 males									
	Mean	SD	Range	Mean	SD	Range	Mean	SD	Range	Test statistics		Group differences				
**Age on day of study**	30.12	4.8	23–40	29.53	5.36	22–39	27.89	4.74	20–37	H_(2,94)_ = 3.09, *p* = 0.21						
**School education (years)**	11.65	0.91	9–13^a^	10.11	1.94	4–12	9.56	2.55	1–12	H_(2,94)_ = 19.83, *p* < 0.01		CON vs. schizophrenia, *p* < 0.01; CON vs. MAP, *p* < 0.01				
**Post-school education (years)**	0.94	1.35	0–4	0.25	0.88	0–5	0.61	1.14	0–4	H_(2,94)_ = 8.44, *p* = 0.02		n.s.				
**Total education duration (years)**	12.58	1.85	9–16^b^	10.37	2.28	4–17	10.17	3.16	1–16	H_(2,94)_ = 18.829, *p* < 0.01		CON vs. schizophrenia, *p* < 0.01; CON vs. MAP, *p* < 0.01				
**Duration of illness (months)**	-	-	-	102.31	52.61	14–228^c^	60	48.34	2–180	*U* = 250, *z* = 3.271, *p* < 0.01						
**No. of psychotic episodes**	-	-	-	3.65	1.62	1–9	2.32	1.08	1–5	*U = 336.5*,* z =-2.07*, *p* = 0.038						
*Antipsychotic use variables*																
**Chlorpromazine equivalents**	-	-	-	337.72	289.02	0-1100	210.74	198.76	0 -750							
**Typical antipsychotics**				*n* = 5			*n* = 6									
**Atypical antipsychotics**				*n* = 21			*n* = 11									
**None**				*n* = 7			*n* = 9									
*Methamphetamine use measures*																
**Duration of use (months)**	-	-	-	64.80	46.16	24–144	104.56	51.64	24–228							
	Mean	SD	Range	Mean	SD	Range	Mean	SD	Range							
**Abstinence (months)**	-	-	-	40.41	46.793	0.03–132	8.01	13.47	0.03–48							
*Psychotic symptomatology PANSS*																
**Positive scale**	7.10	0.41	7–9	12.16	4.88	7–25^d^	10.59	5.23	1–23	H_(2,92)_ = 40.21, *p* < 0.01		CON vs. schizophrenia, *p* < 0.01; CON vs. MAP, *p* < 0.01				
**Negative scale**	7.07	0.26	7–8	14.89	6.41	7– 28^d^	14.30	10.02	7–45	H_(2,92)_ = 43.86, *p* < 0.01		CON vs. schizophrenia, *p* < 0.01; CON vs. MAP, *p* < 0.01				
**General psychopathology**	16.31	0.71	16–19	21.81	7.08	16–45^d^	20.74	6.21	16–37	H_(2,92)_ = 32.99, *p* < 0.01		CON vs. schizophrenia, *p* < 0.01; CON vs. MAP, *p* < 0.01				
**Total score**	30.48	1.12	30–35	48.86	15.56	30–91^e^	45.63	18.68	30–97	H_(2,92)_ = 46.64, *p* < 0.01		CON vs. schizophrenia, *p* < 0.01; CON vs. MAP, *p* < 0.01				

^a^ CON longer duration of school education compared to schizophrenia and MAP

^b^ CON longer duration total education than schizophrenia and MAP

^c^ schizophrenia greater duration of illness compared to MAP

^d^ schizophrenia higher scores than CON and MAP

^e^ schizophrenia lower scores than MAP

Abbreviations: CON– healthy controls; MAP– methamphetamine-induced psychosis. PANSS– Positive and Negative Syndrome Scale; SD– standard deviation

### Subcortical volumes

There were decreased left amygdala volumes in schizophrenia (F_(2,91)_ = 6.37, *p* = 0.012,

$\:{\eta\:\rho\:}^{2}$ = 0.012) and in MAP (F_(2,91)_ = 6.37, *p* = 0.0054, $\:{\eta\:\rho\:}^{2}$ = 0.012) compared to healthy controls (Table 2). There were increased bilateral caudate (F_(2,91)_ = 4.11, *p* = 0.03, $\:{\eta\:\rho\:}^{2}$ = 0.082; F_(2,91)_ = 3.95, *p* = 0.05, $\:{\eta\:\rho\:}^{2}$ = 0.08), putamen (F_(2,91)_ = 6.85, *p* = 0.016, $\:{\eta\:\rho\:}^{2}$ = 0.013; F_(2,91)_ = 5.27, *p* = 0.0072, $\:{\eta\:\rho\:}^{2}$ = 0.10) and NAcc (F_(2,91)_ = 18.69, *p* < 0.0001, $\:{\eta\:\rho\:}^{2}$ = 0.29; F_(2,91)_ = 7.83, *p* = 0.014, $\:{\eta\:\rho\:}^{2}$ = 0.14) volumes in schizophrenia compared to healthy controls. Decreased bilateral NAcc (F_(2,91)_ = 18.69; *p* < 0.0001, $\:{\eta\:\rho\:}^{2}$ = 0.29; F_(2,91)_ = 7.83, *p* = 0.0017, $\:{\eta\:\rho\:}^{2}$ = 0.14) and right globus pallidus (F_(2,91)_ = 3.81, *p* = 0.021, $\:{\eta\:\rho\:}^{2}$ = 0.076) volumes were observed in MAP compared to schizophrenia (Table 2).

**Table 2:** Subcortical volume group differences across schizophrenia, methamphetamine-induced psychosis and healthy controls

Regions	All groups			SCZ-CON	MAP-CON	MAP-SCZ
	Effect size $\:{\eta\:\rho\:}^{2}$	CI: 90%	*p*	*p*	*p*	*p*
Left hippocampus	0.012	[0, 0.056]	0.57	n.s.	n.s.	n.s.
Right hippocampus	0.051	[0, 0.13]	0.090	n.s.	n.s.	n.s.
Left amygdala	0.012	[0.028, 0.22]	0.0026	0.012	0.054	n.s.
Right amygdala	0.029	[0, 0.091]	0.26	n.s.	n.s.	n.s.
Left caudate	0.082	[0.0075, 0.17]	0.020	0.03	n.s.	n.s.
Right caudate	0.08	[0.0062, 0.17]	0.023	0.05	n.s.	n.s.
Left putamen	0.13	[0.033, 0.23]	0.0017	0.016	n.s.	n.s.
Right putamen	0.10	[0.017, 0.2]	0.0068	0.0072	n.s.	n.s.
Left globus pallidus	0.035	[0, 0.1]	0.19	n.s.	n.s.	n.s.
Right globus pallidus	0.076	[0.052, 0.16]	0.026	n.s.	n.s.	0.021
Left nucleus accumbens	0.29	[0.16, 0.39]	< 0.0001	< 0.0001	n.s.	< 0.0001
Right nucleus accumbens	0.14	[0.043, 0.25]	0.0007	0.014	n.s.	0.0017

Effect size: for all group differences. *MANOVA (F(2, 91)) was performed for subcortical volumes in methamphetamine-induced psychosis (MAP), schizophrenia (SCZ) and healthy controls (CON) groups, with *p*-values of < 0.05 considered statistically significant. n.s: non-significant. Effect sizes: partial eta squared (Confidence Interval: 90%)

No significant differences in subcortical volumes were observed between the schizophrenia group which reported methamphetamine use and the schizophrenia group who did not report methamphetamine use (Supplementary Table 1).

There were increased left nucleus accumbens volumes in the MAP participants treated with atypical antipsychotics versus schizophrenia participants treated with atypical antipsychotics (F_(5, 53)_ = 8.17, *p* = 0.032, $\:{\eta\:\rho\:}^{2}$ = 0.44), between the MAP participants treated with typical antipsychotics versus schizophrenia group on atypical antipsychotics (F_(5, 53)_ = 8.17, *p* = 0.001, $\:{\eta\:\rho\:}^{2}$ = 0.44), between antipsychotic naïve MAP versus schizophrenia participants on atypical antipsychotics (F_(5, 53)_ = 8.17, *p* = 0.0013, $\:{\eta\:\rho\:}^{2}$ = 0.44), MAP participants treated with atypical antipsychotics versus antipsychotic naïve schizophrenia participants (F_(5, 53)_ = 8.17, *p* = 0.046, $\:{\eta\:\rho\:}^{2}$ = 0.44), MAP participants treated with typical antipsychotics versus antipsychotic naïve schizophrenia participants (F_(5, 53)_ = 8.17, *p* = 0.016, $\:{\eta\:\rho\:}^{2}$ = 0.44) and in antipsychotic naïve MAP participants and antipsychotic naïve schizophrenia participants (F_(5, 53)_ = 8.17, *p* = 0.0077, $\:{\eta\:\rho\:}^{2}$ = 0.44) (Supplementary Tables 4 and 5).

### Frontal cortical thickness

There was decreased frontal cortical thickness in schizophrenia compared to healthy controls for all frontal regions (F_(2,91)_ > 10.5, *p* < 0.0004): left and right caudal middle frontal (F_(2,91)_ = 36.2, *p* < 0.0001, $\:{\eta\:\rho\:}^{2}$ = 0.44; F_(2,91)_ = 17.9 *p* < 0.0001, = 0.28), left lateral orbitofrontal (H_(2,94)_ = 60.76 *p* < 0.0001, $\:{\epsilon\:}^{2}$= 0.65), left rostral middle frontal (F_(2,91)_ = 40, *p* < 0.0001, $\:{\eta\:\rho\:}^{2}$ = 0.47), left and right superior frontal (F_(2,91)_ = 10.5, *p* < 0.0001, $\:{\eta\:\rho\:}^{2}$ = 0.19; F_(2,91)_ = 4.3, *p* = 0.015, $\:{\eta\:\rho\:}^{2}\:$= 0.085), left and right frontal pole (H_(2,94)_ = 82.3, *p* < 0.0001, $\:{\epsilon\:}^{2}$ = 0.88; H_(2,94)_ = 64.36, *p* < 0.0001, $\:{\epsilon\:}^{2}$ = 0.69), left and right pars opercularis (F_(2, 91)_ = 82.5, *p* < 0.0001, $\:{\eta\:\rho\:}^{2}\:$ = 0.64; F_(2, 91)_ = 31.6, *p* < 0.0001, $\:{\eta\:\rho\:}^{2}$ = 0.41), left and right pars orbitalis (F_(2, 91)_ = 10.5, *p* < 0.0001, $\:{\eta\:\rho\:}^{2}$ = 0.19; F_(2, 91)_ = 13.9 *p* < 0.0001, $\:{\eta\:\rho\:}^{2}\:$ = 0.23) and left and right pars triangularis (F_(2, 91)_ = 30.18, *p* < 0.0001, $\:{\eta\:\rho\:}^{2}\:$ = 0.39; H_(2,94)_ = 29.2 *p* < 0.0004, $\:{\epsilon\:}^{2}$ = 0.31;), except for the right lateral orbitofrontal, left and right medial orbitofrontal and right rostral middle frontal regions (Table 3).

**Table 3:** Frontal cortical thickness group differences across schizophrenia, methamphetamine-induced psychosis and healthy controls

Regions	All groups			SCZ-CON	MAP-CON	MAP-SCZ
	Effect size^*#^	CI: 90%	*p*	*p*	*p*	*p*
Left caudal middle frontal*	0.44	[0.31, 0.53]	< 0.0001	< 0.0001	< 0.0001	n.s.
Right caudal middle frontal*	0.28	[0.15, 0.39]	< 0.0001	< 0.0001	< 0.0001	n.s.
Left lateral orbitofrontal^#^	0.65	[0.64, 065]	< 0.0001	< 0.0001	n.s.	< 0.0001
Right lateral orbitofrontal*	0.19	[0.074, 0.29	< 0.0001	n.s.	0.028	< 0.0001
Left medial orbitofrontal^#^	0.11	[0.11, 0.12]	0.022	n.s.	n.s.	0.0014
Right medial orbitofrontal^#^	0.077	[0.05, 0.064]	0.027	n.s.	0.042	n.s.
Left rostral middle frontal*	0.47	[0.34, 0.56]	< 0.0001	< 0.0001	< 0.0001	n.s.
Right rostral middle frontal^#^	0.096	[0.091, 0.10]	0.0046	n.s.	n.s.	0.0032
Left superior frontal*	0.19	[0.071, 0.29]	< 0.0001	< 0.0001	0.013	n.s.
Right superior frontal*	0.085	[0.0091, 0.18]	0.016	0.015	n.s.	n.s.
Left frontal pole^#^	0.88	[0.87, 0.89]	< 0.0001	< 0.0001	0.0002	< 0.0001
Right frontal pole^#^	0.69	[0.67, 0.70]	< 0.0001	< 0.0001	< 0.0001	n.s.
Left pars opercularis^*^	0.64	[0.54, 0.71]	< 0.0001	< 0.0001	< 0.0001	< 0.0001
Right pars opercularis^*^	0.41	[0.27, 0.5]	< 0.0001	< 0.0001	< 0.0001	0.0001
Left pars orbitalis^*^	0.19	[0.071, 0.29]	< 0.0001	< 0.0001	0.0068	n.s.
Right pars orbitalis^*^	0.23	[0.11, 0.34]	< 0.0001	< 0.0001	0.0002	n.s.
Left pars triangularis^*^	0.39	[0.25, 0.49]	< 0.0001	< 0.0001	n.s.	< 0.0001
Right pars triangularis^#^	0.31	[0.30, 0.32]	< 0.0001	0.0004	n.s.	< 0.0001

Effect size for all group differences. *MANOVA (F(2,91)) and #Kruskal Wallis ANOVA (H(2,94)) performed for frontal cortical thickness in methamphetamine-induced psychosis (MAP), schizophrenia (SCZ) and healthy controls (CON) groups, with *p*-values of < 0.05 considered statistically significant. n.s: results which did not survive the MANOVA and Kruskal Wallis ANOVA. Effect sizes, *partial eta squared (Confidence Interval: 90%) and #epsilon squared (Confidence Interval: 90%)

Decreased frontal cortical thickness in MAP compared to healthy controls for all frontal regions (All, F_(2,91)_ > 10.5, *p* < 0.013) was found: left and right caudal middle frontal (F_(2,91)_ = 36.2, *p* < 0.0001, $\:{\eta\:\rho\:}^{2}$ = 0.44; F_(2,91)_ = 17.9, *p* < 0.0001, $\:{\eta\:\rho\:}^{2}$ = 0.28), left rostral middle frontal (F_(2,91)_ = 40, *p* < 0.0001, $\:{\eta\:\rho\:}^{2}$ = 0.47), left superior frontal (F_(2,91)_ = 10.5, *p* = 0.013, $\:{\eta\:\rho\:}^{2}$ = 0.085), left and right frontal pole (H_(2,94)_ = 82.3 *p* < 0.0001, $\:{\epsilon\:}^{2}$ = 0.88); H_(2,94)_ = 64.36, *p* = 0.0002, $\:{\epsilon\:}^{2}$ = 0.69) left and right pars opercularis (F_(2, 91)_ = 82.5, *p* < 0.0001, $\:{\eta\:\rho\:}^{2}$ = 0.64; F_(2, 91)_ = 31.6, *p* < 0.0001, $\:{\eta\:\rho\:}^{2}$ = 0.41) and the left and right pars orbitalis (F_(2, 91)_ = 10.5, *p* = 0.0068, $\:{\eta\:\rho\:}^{2}$ = 0.19; F_(2, 91)_ = 13.9 *p* = 0.0002, $\:{\eta\:\rho\:}^{2}$ = 0.23), except for the left lateral orbitofrontal, medial orbitofrontal, right rostral middle frontal and superior frontal and the left and right pars triangularis, and increased cortical thickness in MAP compared to healthy controls in the right medial orbitofrontal (H_(2,94)_ = 7.21, *p* = 0.027, $\:{\epsilon\:}^{2}$ = 0.077) and right lateral orbitofrontal (F_(2,91)_ = 10.7, *p* = 0.028, $\:{\eta\:\rho\:}^{2}$ = 0.019) (Table 3).

Increased frontal cortical thickness was observed in MAP compared to schizophrenia for the right lateral orbitofrontal (F_(2,91)_ = 10.7, *p* < 0.0001, $\:{\eta\:\rho\:}^{2}$ = 0.65), left pars opercularis (F_(2,91))_ = 82.5, *p* < 0.0001, $\:{\eta\:\rho\:}^{2}\:$= 0.64), left lateral orbitofrontal (H_(2,94)_ = 60.76, *p* < 0.0001, $\:{\epsilon\:}^{2}$ = 0.65), left medial orbitofrontal (H_(2,94)_ = 12.22, *p* = 0.0014, $\:{\epsilon\:}^{2}\:$= 0.11), left frontal pole (H_(2,94)_ = 82.30, *p* < 0.0001, $\:{\epsilon\:}^{2}$ = 0.88) and left and right pars triangularis (F_(2,91)_ = 29.2, *p* < 0.0001, = 0.39 and $\:{\eta\:\rho\:}^{2}$ = 0.31). There was increased frontal cortical thickness was observed in schizophrenia compared to MAP for the right rostral middle frontal (H_(2,94)_ = 10.78, *p* = 0.0032, $\:{\epsilon\:}^{2}$ = 0.096) and the right pars opercularis (F_(2,91)_ = 31.6, *p* = 0.0001, $\:{\eta\:\rho\:}^{2}$ = 0.41) (Table 3).

Increased frontal cortical thickness was observed in schizophrenia group which used methamphetamine for the left lateral orbitofrontal (*U* = 64.5, *p* = 0.039, = 0.47) and right medial orbitofrontal (*U* = 67.5, *p* = 0.05, = 0.44) compared to the schizophrenia group who reported no methamphetamine use. Decreased frontal cortical thickness was observed in the schizophrenia group who reported methamphetamine use for the left frontal pole (*U* = 178.5, *p* = 0.039, = -0.47) compared to the schizophrenia group who reported no methamphetamine use (Supplementary Tables 2 and 3).

Increased left lateral orbitofrontal cortical thickness was observed in MAP participants on atypical antipsychotics versus schizophrenia participants on atypical antipsychotics (H_(5,59)_ = 37.13, *p* < 0.0001, $\:{\epsilon\:}^{2}$ = 0.61), MAP participants on typical antipsychotics versus schizophrenia participants on typical antipsychotics (H_(5,59)_ = 37.13, *p* = 0.029, $\:{\epsilon\:}^{2}$ = 0.61), MAP participants on atypical antipsychotics versus schizophrenia on typical antipsychotics (H_(5,59)_ = 37.13, *p* = 0.0038, $\:{\epsilon\:}^{2}$ = 0.61), antipsychotic naïve MAP participants and schizophrenia on atypical and typical antipsychotics (H_(5,59)_ = 37.13, *p* = 0.0029, $\:{\epsilon\:}^{2}$ = 0.61 and *p* < 0.027). Increased right lateral orbitofrontal was observed in the MAP participants on atypical antipsychotics versus schizophrenia participants on atypical antipsychotics (F_(5, 53)_ = 5.46, *p* = 0.042, $\:{\eta\:\rho\:}^{2}$ = 0.34) (Supplementary Tables 6 and 7).

Increased left frontal pole was observed in the MAP participants on atypical antipsychotics versus the schizophrenia participants on atypical antipsychotics (H_(5,59)_ = 44.24, *p* = 0.00036, $\:{\epsilon\:}^{2}$ = 0.74), MAP participants on typical antipsychotics versus schizophrenia participants on atypical antipsychotics (H_(5,59)_ = 44.24, *p* = 0.0025, $\:{\epsilon\:}^{2}$ = 0.74), between antipsychotic naïve MAP participants versus schizophrenia participants on atypical antipsychotics (H_(5,59)_ = 44.24, *p* = 0.00012, $\:{\epsilon\:}^{2}$ = 0.74), between MAP participants on atypical antipsychotics and antipsychotic naïve schizophrenia participants (H_(5,59)_ = 44.24, *p* = 0.0012, $\:{\epsilon\:}^{2}$ = 0.74), between MAP participants on typical antipsychotics and antipsychotic naïve schizophrenia participants (H_(5,59)_ = 44.24, *p* = 0.0028, $\:{\epsilon\:}^{2}$ = 0.74) and between antipsychotic naïve MAP participants versus antipsychotic naïve schizophrenia participants (H_(5,59)_ = 44.24, *p* = 0.0004, $\:{\epsilon\:}^{2}$ = 0.74) (Supplementary Tables 6 and 7).

Increased left pars triangularis was observed between MAP participants on atypical antipsychotics versus schizophrenia participants on atypical antipsychotics (F_(5, 53)_ = 10.83, *p* = 0.0003, $\:{\eta\:\rho\:}^{2}$ = 0.51), between MAP participants on typical antipsychotics versus schizophrenia participants on atypical antipsychotics (F_(5, 53)_ = 10.83, *p* = 0.0034, $\:{\eta\:\rho\:}^{2}$ = 0.51), between antipsychotic naïve MAP participants and schizophrenia participants in atypical antipsychotics (F_(5, 53)_ = 10.83, *p* < 0.0001, $\:{\eta\:\rho\:}^{2}$ = 0.51) (Supplementary Tables 6 and 7).

Increased right pars triangularis was observed in the MAP participants on atypical antipsychotics versus the schizophrenia participants on atypical antipsychotics (H_(5,59)_ = 28.77, *p* = 0.001 $\:{\epsilon\:}^{2}$ = 0.45), MAP participants on typical antipsychotics versus the schizophrenia participants on atypical antipsychotics (H_(5,59)_ = 28.77, *p* = 0.03, $\:{\epsilon\:}^{2}$ = 0.45), MAP participants on atypical antipsychotics and schizophrenia participants on typical antipsychotics (H_(5,59)_ = 28.77, *p* = 0.015, $\:{\epsilon\:}^{2}$ = 0.45), antipsychotic naïve MAP participants and schizophrenia participants on atypical (H_(5,59)_ = 28.77, *p* = 0.013, $\:{\epsilon\:}^{2}$ = 0.45) and typical (H_(5,59)_ = 28.77, *p* = 0.054, $\:{\epsilon\:}^{2}$ = 0.45) antipsychotics (Supplementary Tables 6 and 7).

### Cytokines

There were no significant group differences in cytokine concentrations for IFN$\:\gamma\:$ (H_2,94_ = 2.81; *p* = 0.245, $\:{\epsilon\:}^{2}$ = 0.039), IL-10 (H_2,94_ = 0.93, *p* = 0.627, $\:{\epsilon\:}^{2}$ = 0.0099), IL-12 (H_2,94_ = 4.90, *p* = 0.0862, $\:{\epsilon\:}^{2}$ = 0.011), IL-1(H_2,94_ = 1.27, *p* = 0.531, $\:{\epsilon\:}^{2}$ = 0.014), IL-8 (H_2,94_ = 0.23; *p* = 0.892, $\:{\epsilon\:}^{2}$ = 0.0024) and TNF-$\:\alpha\:$ (H_2,94_ = 1.01, *p* = 0.605, $\:{\epsilon\:}^{2}$ = 0.011).

### Associations of neuroimaging measures with pro-inflammatory cytokines, illness duration and symptom severity

There were no significant associations between neuroimaging measures and either symptom severity with cytokine concentrations.

In schizophrenia, longer illness duration was associated with decreased left amygdala volumes (R_(*n*=36)_ = -0.43, *p* = 0.0088) and decreased left and right caudal middle frontal (R_(*n*=36)_ = -0.64, *p* < 0.0001; R_(*n*=36)_ = -0.64, *p* < 0.0001), left and right lateral orbitofrontal (R_(*n*=36)_ = -0.52, *p* = 0.0013; R_(*n*=36)_ = -0.62, *p* < 0.0001), left and right medial orbitofrontal (R_(*n*=36)_ = -0.58, *p* < 0.0001; R_(*n*=36)_ = -0.55, *p* < 0.0001), left and right rostral middle frontal (R_(*n*=36)_ = -0.6, *p* < 0.0001; R_(*n*=36)_ = -0.57, *p* = 0.0001), left and right superior frontal (R_(*n*=36)_ = -0.59, *p* = 0.00014; R_(*n*=36)_ = -0.63, *p* < 0.0001), left and right pars opercularis (R_(*n*=36)_ = -0.65, *p* < 0.0001; R_(*n*=36)_ = -0.63, *p* < 0.0001), left and right pars orbitalis (R_(*n*=36)_ = -0.64, *p* < 0.0001; R_(*n*=36)_ = -0.65, *p* < 0.0001) left and right pars triangularis (R_(*n*=36)_ = -0.58, *p* = 0.0002; R_(*n*=36)_ = -0.65, *p* < 0.0001) and increased left frontal pole (R_(*n*=36)_ = 0.48, *p* = 0.0029) However, with a threshold of *p* < 0.05, decreased illness duration was associated with increased left caudate (R_(*n*=36)_ = -0.43; *p* = 0.0084), right caudate (R_(*n*=36)_ = -0.45; *p* = 0.0061), left putamen (R_(*n*=36)_ = -0.48; *p* = 0.0032), right putamen (R_(*n*=36)_ = -0.44; *p* = 0.0066), left pallidus (R_(*n*=36)_ = -0.35; *p* = 0.039), left NAcc (R_(*n*=36)_ = -0.46; *p* = 0.0050) and right NAcc (R_(*n*=36)_ = -0.45; *p* = 0.0054).

In MAP, increased duration of methamphetamine use was associated with decreased left amygdala (r_(*n*=27)_ = -0.51, *p* = 0.0069) volumes however, with a threshold of *p* < 0.05 we found an association with decreased right amygdala (r_(*n*=27)_ = -0.47, *p* = 0.012). Increased duration of methamphetamine use was also associated with decreased left lateral orbitofrontal (R_(*n*=27)_ = -0.54, *p* = 0.0030), left medial orbitofrontal (R_(*n*=27)_ = -0.55; *p* = 0.0030), right medial orbitofrontal (R_(*n*=27)_ = -0.55, *p* = 0.0030), right rostral middle frontal (R_(*n*=27)_ = -0.55, *p* = 0.0030) and left frontal pole (R_(*n*=27)_ = -0.55, *p* = 0.0030). However, with a threshold of *p* < 0.05, increased duration of methamphetamine use was associated with decreased left caudal middle frontal (r_(*n*=27)_ = -0.43, *p* = 0.025) and right lateral orbitofrontal (r_(*n*=27)_ = 0.46, *p* = 0.016) cortices. In MAP, at a threshold of *p* < 0.05, decreased duration of methamphetamine use was associated with increased left caudate (r_(*n*=27)_ = -0.44, *p* = 0.021), left and right putamen (r_(*n*=27)_ = -0.38, *p* = 0.05; r_(*n*=27)_ = -0.43, *p* = 0.025), left globus pallidus (r_(*n*=27)_ = -0.41, *p* = 0.032) and right nucleus accumbens volumes (r_(*n*=27)_ = -0.40, *p* = 0.039).

## Discussion

The findings of this study include decreased left amygdala volumes and frontal cortical thickness in schizophrenia and MAP compared to controls; increased volumes in bilateral caudate, putamen, and nucleus accumbens (NAcc) volumes in schizophrenia compared to controls; and increased right globus pallidus and bilateral NAcc volumes in schizophrenia compared to MAP. There were no significant differences across groups for cytokine levels and their associations with neuroimaging measures. Lastly, decreased left amygdala volumes and frontal cortical thickness were significantly associated with longer illness duration in schizophrenia and with longer duration of methamphetamine use in MAP.

The present data are consistent with previous findings that decreased left amygdala volumes and frontal cortical thinning are present in both schizophrenia (Niu et al., 2004; van Erp et al., 2016) and MAP (Orikabe et al., 2011; Uhlmann et al., 2016) compared to healthy controls, and are associated with duration of illness in schizophrenia (Bustillo et al., 2001; Molina et al., 2011). This study is the first to our knowledge showing decreased amygdala volume and frontal cortical thickness in MAP. Taken together, these findings suggest important overlaps in the neurobiology of schizophrenia and MAP. Decreased cortical thickness and thus pyramidal neuron layers may reflect decreased neural density and functional dysconnectivity to subcortical and other cortical brain regions, which has been reported to underlie the symptoms of schizophrenia (Lewis et al., 2012; Li et al., 2016, 2019) and therefore may occur in MAP. However, further studies are needed to elucidate whether psychotic symptoms in MAP are indeed due to loss of neural connectivity from the frontal cortical neurons to subcortical and cortical structures.

Increased caudate volume was observed in schizophrenia but not in MAP, which is a consistent finding in schizophrenia and has been attributed to typical antipsychotic use (Breier et al., 1992; Chakos et al., 1994; Emsley et al., 2023; Jørgensen et al., 2016; Lang et al., 2004). The finding of increased basal ganglia volumes has been replicated in several studies, including putamen volumes in chronic schizophrenia (Emsley et al., 2023; Hokama et al., 1995; Kitabayashi et al., 2006; Mamah et al., 2007; van Erp et al., 2016). The finding that globus pallidus and NAcc volumes were increased in schizophrenia compared with MAP is a novel one, and may also highlight important differences in the neurobiology between schizophrenia and MAP. However, it is notable that increased globus pallidus volume was associated with increased duration of illness in schizophrenia, and that increased volume of these structures has been attributed to antipsychotic use. Given that use of antipsychotic agents is typically of lower duration in MAP, we cannot exclude the possibility that this finding points to differences in treatment duration between schizophrenia and MAP. Secondly, since the basal ganglia volumes in MAP were not significantly different to those in healthy controls, this could suggest partial gray matter recovery due to methamphetamine abstinence, which has been reported to reduce oxidative stress and in turn neuroinflammation, in abstinent methamphetamine abusers (Huang et al., 2013).

Decreased frontal cortical thickness was observed in the superior, middle and inferior frontal brain regions in schizophrenia compared to healthy controls, in addition to that of the left lateral OFC, widely reported finding in schizophrenia (Ahmed et al., 2015; Narr et al., 2005; Rimol et al., 2010, 2012; Sprooten et al., 2013; Uhlmann et al., 2016; van Erp et al., 2018; van Haren et al., 2011; Walton et al., 2018; Xiao et al., 2015; Zugman et al., 2013), this was also observed for the schizophrenia group who did not report methamphetamine use. Cortical thickness has been reported to decrease more rapidly throughout the course of the illness (DeLisi et al., 1997; van Haren et al., 2011) compared to healthy controls and has also been observed in chronic schizophrenia (Goldman et al., 2009; Kuperberg et al., 2003; Madre et al., 2020; Nesvag et al., 2008). In MAP we observed significant decreases for the right medial OFC and lateral OFC cortical thickness compared to healthy controls, whilst the schizophrenia group showed significant decreases in cortical thickness of the lateral OFC and left medial OFC compared to MAP, suggesting a deleterious effect of schizophrenia on these regions in the left hemisphere. However, the sensitivity analysis suggests that these findings were not observed in the schizophrenia group who reported no methamphetamine use, therefore this finding may be attributed to methamphetamine use instead of the structural differences we observed between the two psychotic disorders. This finding suggests decreased frontal cortical thickness in the right hemisphere in MAP compared to schizophrenia, occurring in the left hemisphere, which may aid in distinguishing MAP from schizophrenia. This is the first study to report decreased cortical thickness of the OFC regions in MAP, which has been reported in antipsychotic naïve schizophrenia individuals (Venkatasubramanian et al., 2008). We observed increased left frontal pole in the schizophrenia group compared to MAP as well as in the schizophrenia group who did not report methamphetamine use compared to those who reported methamphetamine use.

In MAP we observed significant decreases for the right lateral and medial OFC cortical thickness compared to healthy controls, whilst the schizophrenia group showed significant decreases in cortical thickness compared to that of MAP, particularly the lateral and left medial OFC. We speculate that the considerable duration of illness in the schizophrenia participants of this study may contribute to the differences between schizophrenia to MAP. In this respect, longer illness duration has been attributed to cortical thinning in patients with schizophrenia, including the PFC (DeLisi et al., 1997; van Haren et al., 2011). However, more recent literature has reported decreased cortical thickness in schizophrenia patients treated with antipsychotics compared to healthy controls (van Erp et al., 2018), and therefore this could be a plausible explanation for decreased cortical thickness in the schizophrenia group in the current study. Our results also showed that longer duration of methamphetamine use was significantly associated with decreased frontal cortical thickness in MAP. Further research is needed to determine whether schizophrenia has a distinct deleterious effect on the pathophysiology of reduced cortical thickness in the lateral and medial OFC regions in the left hemisphere, or whether these brain regions are also affected with longer duration of methamphetamine use in MAP. Longer duration of methamphetamine use was also associated with decreased left caudate, left and right putamen, left globus pallidus and right NAcc volumes, in MAP participants. This is consistent with a study by Chang et al., 2005, who found decreased putamen and globus pallidus volumes to be associated with increased lifetime duration of methamphetamine use, suggesting a possible loss of basal ganglia volumes with greater methamphetamine use (Chang et al., 2005).

Decreased frontal cortical thickness in the schizophrenia participants treated with atypical antipsychotics compared to MAP subgroups could be attributed to the anti-inflammatory properties elicited by atypical antipsychotics, which has previously been reported in schizophrenia (Juncal-Ruiz et al., 2018; Miller et al., 2011; Sobiś et al., 2015). Clozapine, an atypical antipsychotic, has been shown to suppress proinflammatory cytokine production in mice, whilst increasing anti-inflammatory cytokines (Sugino et al., 2009). In support of this, a previous study reported significant cortical thinning of the medial prefrontal cortex in schizophrenia patients treated with clozapine, although this was in comparison to healthy controls (Ahmed et al., 2015), which could be attributed to the anti-inflammatory effects of clozapine. However, it remains unclear whether atypical antipsychotics exert similar anti-inflammatory effects in MAP or whether the anti-inflammatory effects of atypical antipsychotics are ameliorated by the neurotoxicity of methamphetamine, highlighting the need for larger studies to explore this potential mechanism. Furthermore, the findings from the antipsychotic sensitivity analysis suggest that the initial group-level ANOVA, which classified participants based on diagnosis alone, may have obscured the more nuanced structural differences related to antipsychotic treatment. By accounting for medication subgroups, we identified significant differences that were not inherently phenotypical in origin, suggesting that the variability in brain structure may be influenced by the treatment response rather than solely by diagnostic classification. To our knowledge, this is the first study to investigate the effects of antipsychotic treatment on frontal cortical thickness and subcortical volumes in individuals diagnosed with MAP, emphasising the need for future research to disentangle medication effects from underlying disease pathology.

We found no significant differences in cytokine concentrations across groups. Furthermore, no significant associations were observed between neuroimaging measures and cytokines levels. There is some inconsistency in the literature on immune markers on schizophrenia; our findings are consistent with some previous work (Kaminska et al., 2001; Lv et al., 2015; Miller et al., 2011; Potvin et al., 2008; Wilke et al., 1996). However, we cannot rule out the possibility that our negative findings are due to insufficient power. Moreover, the anti-inflammatory effects of antipsychotic medication in both schizophrenia and MAP groups may have ameliorated the inflammatory status in these study groups (Flatow et al., 2013; Fraguas et al., 2019; Miller et al., 2011). Furthermore, antipsychotic treatment has also been shown to suppress microglial activation (Bian et al., 2008; Pillai et al., 2006), which has been implicated in the pathophysiology of schizophrenia and may have ameliorated the neuroinflammatory profiles we expected to find in this study.

A number of limitations deserve emphasis. First, the use of antipsychotics in both schizophrenia and MAP patients may have confounded the results reported in this study, as the effects of antipsychotic treatment on brain structure is heterogenous in nature and is dependent on individual patient response. Given that antipsychotic exposure was not controlled for, it would be difficult to determine whether the observed differences in brain volume and cortical thickness are due to the disorders themselves or the treatment. Second, data for antipsychotic lifetime dose or years of exposure were not collected during this study, therefore this study was unable to determine whether these were associated with brain volume and cortical thickness measures. Third, although our sample size is consistent with previous work on MAP, the small sample size warrants replication in a larger study and findings may thus reflect random variation rather than true effects. Strategies to improve the sample size in future studies could include a longer duration of patient recruitment, partnering with other institutions from multiple locations within the country or internationally and leveraging existing neuroimaging datasets. In addition, rare but clinically relevant patterns may go undetected. Fourth, self-reported methamphetamine abstinence was not supported by an objective confirmation. Fifth, sex differences were not examined due to the small sample of female participants and thus results may be obscured and lead to biased interpretations. Sixth, methamphetamine use in some participants in the schizophrenia group may have confounded the comparisons with the MAP group, however our sensitivity analysis provides some reassurance that this is not the case. Seventh, due to the small subgroup size in the sensitivity analysis for methamphetamine use in the schizophrenia group, the robustness of the reassurance provided by the sensitivity analysis may be limited. Eighth, the lack of significant cytokine differences across the three groups may be due to the small sample size which may have been insufficient to detect small cytokine differences. Due to the biological nature of cytokines, concentrations are sensitive to stress, illness and circadian rhythms which could result in variations in their concentrations. Additionally, cytokine elevations may also occur in some endophenotypes and not in others and lastly many of the schizophrenia and MAP patients were on atypical antipsychotics which have been reported to elicit anti-inflammatory properties, which may have masked neuroinflammation which may be diagnosis-specific. Lastly, cytokine elevations may not always present due to the selective permeability of the blood brain barrier, in addition no cerebrospinal fluid was collected and analysed for cytokine concentrations due to the invasiveness of obtaining cerebrospinal fluid samples, however, this could be addressed in future studies. Ninth, the adjustment of Rho and alpha threshold values, in a small sample study such as the current study, may miss the detection of stronger correlations which do not meet the stricter alpha threshold even though the effect is real and thus goes unreported.

### Conclusions

This study shows distinctive structural variations in basal ganglia and amygdala volumes, in addition to frontal cortical thickness, in individuals with schizophrenia and MAP, suggesting that these structures play a pivotal role in the pathophysiology of these disorders. Differences in basal ganglia volumes between schizophrenia and MAP may be attributed by longer illness duration in schizophrenia. The novel discovery of increased globus pallidus and NAcc volumes in schizophrenia compared with MAP may show crucial distinctions in the neurobiology between these two conditions. To deepen our understanding of the neurobiological underpinnings of MAP, future investigations should employ larger sample sizes in order to accurately reflect the true effects of MAP on brain structure in addition to incorporating lifetime methamphetamine exposure. Secondly, findings in the MAP group may benefit from a longitudinal study investigating the initial abuse of MA through to the diagnosis of MAP before treatment with antipsychotics, through to antipsychotic treated MAP, to determine the effects of each variable on brain structure and cytokine profiles in MAP. Thirdly, in order examine the true effects of neuroinflammation on brain regions in both schizophrenia and MAP, future studies could employ magnetic resonance imaging, which would provide valuable insight on neuronal viability and the possible effects of antipsychotics and methamphetamine-induced neurotoxicity on brain structure, through the analysis of neurometabolites.

## Electronic supplementary material

Below is the link to the electronic supplementary material.

Supplementary Material 1

## Data Availability

No datasets were generated or analysed during the current study.
