# Supplementary material for: Subcortical volumes, frontal cortical thickness, and pro-inflammatory cytokines in schizophrenia versus methamphetamine-induced psychosis
Source: Brain Imaging Behav. 2025 May 28;19(4):874–88. doi: 10.1007/s11682-025-01022-9 (PMC12310862; doi:10.1007/s11682-025-01022-9)
Supplement: Supplementary file 1 — Supplementary Material 1 [file 11682_2025_1022_MOESM1_ESM.docx]

**Supplementary Material**

Manuscript title: *Subcortical volumes, frontal cortical thickness, and pro-inflammatory cytokines in schizophrenia versus methamphetamine-induced psychosis*

Supplementary Table 1: Subcortical volume group differences across schizophrenia with and without methamphetamine use

| Regions | Effect size  Cohen’s d | CI: 95% | *p* | Effect size  Hedges’ g | t-value |
| --- | --- | --- | --- | --- | --- |
| Left hippocampus | 0.35 | [-0.35, 1.04] | 0.26 | 0.34 | 1.02 |
| Right hippocampus | 0.32 | [-0.38, 1.01] | 0.31 | 0.31 | 0.93 |
| Left amygdala | 0.22 | [-0.47, 0.91] | 0.50 | 0.21 | 0.64 |
| Right amygdala | 0.28 | [-0.41, 0.98] | 0.37 | 0.28 | 0.83 |
| Left caudate | 0.17 | [-0.53, 0.86] | 0.61 | 0.16 | 0.49 |
| Right caudate | 0.06 | [-0.63, 0.76] | 0.85 | 0.06 | 0.18 |
| Left putamen | 0.03 | [-0.66, 0.73] | 0.92 | 0.03 | 0.10 |
| Right putamen | 0.13 | [-0.57, 0.82] | 0.70 | 0.12 | 0.37 |
| Left globus pallidus | 0.18 | [-0.52, 0.87] | 0.59 | 0.17 | 0.52 |
| Right globus pallidus | 0.20 | [-0.50, 0.89] | 0.53 | 0.20 | 0.58 |
| Left nucleus accumbens | 0.06 | [-0.64, 0.75] | 0.86 | 0.06 | 0.17 |
| Right nucleus accumbens | 0.08 | [-0.61, 0.78] | 0.80 | 0.08 | 0.25 |
| Effect size for all group differences. Parametric independent samples t-test performed for subcortical volumes in schizophrenia with methamphetamine use (SCZ_MA) and without methamphetamine use (SCZ), with *p*-values of <0.05 considered statistically significant. Effect sizes, *Cohens d at 95% confidence interval. | | | | | |

Supplementary Table 2 Frontal cortical thickness group differences across schizophrenia with and without methamphetamine use

| Regions | Effect size  Cohen’s d | CI: 95% | *p* | Effect size  Hedges’ g | t-value |
| --- | --- | --- | --- | --- | --- |
| Left caudal middle frontal | 0.56 | [-0.22, 1.35] | 0.13 | 0.55 | 1.46 |
| Right caudal middle frontal | 0.66 | [-0.22, 1.35] | 0.07 | 0.64 | 1.7 |
| Right lateral orbitofrontal | 0.71 | [-0.07, 1.49] | 0.045 | 0.69 | 1.84 |
| Left rostral middle frontal | 0.19 | [-0.59, 0.97] | 0.59 | 0.19 | 0.49 |
| Left superior frontal | 0.15 | [-0.63, 0.94] | 0.66 | 0.15 | 0.4 |
| Right superior frontal | 0.29 | [-0.49, 1.07] | 0.43 | 0.28 | 0.43 |
| Left pars opercularis | 0.54 | [-0.24, 1.32] | 0.15 | 0.53 | 1.4 |
| Right pars opercularis | 0.67 | [-0.11, 1.45] | 0.063 | 0.66 | 1.74 |
| Left pars orbitalis | 0.35 | [-0.43, 1.13] | 0.34 | 0.34 | 0.90 |
| Right pars orbitalis | 0.43 | [-0.35, 1.21] | 0.25 | 0.42 | 1.12 |
| Left pars triangularis | 0.07 | [-0.72, 0.85] | 0.85 | 0.06 | 0.17 |
| Effect size for all group differences. Parametric independent samples t-test performed for frontal cortical thickness in schizophrenia with methamphetamine use (SCZ_MA) and without methamphetamine use (SCZ), with *p*-values of <0.05 considered statistically significant.. Effect sizes, *Cohens d at 95% confidence interval. | | | | | |

Supplementary Table 3: Frontal cortical thickness group differences across schizophrenia with and without methamphetamine use

| Regions | Biserial Correlation Coefficient  *r* | CI: 95% | *p* | U |
| --- | --- | --- | --- | --- |
| Left lateral orbitofrontal | 0.47 | [0.041, 0.34] | 0.039 | 64.5 |
| Left medial orbitofrontal | 0.44 | [0.054, 0.32] | 0.055 | 68.5 |
| Right medial orbitofrontal | 0.44 | [0.047, 0.33] | 0.050 | 67.5 |
| Right rostral middle frontal | 0.44 | [0.048, 0.33] | 0.054 | 68.5 |
| Left frontal pole | -0.47 | [0.064, -0.31] | 0.039 | 178.5 |
| Right frontal pole | 0.12 | [0.10, 0.28] | 0.6 | 106.5 |
| Right pars triangularis | 0.25 | [0.20, 0.24] | 0.27 | 91 |
| Effect size for all group differences. Non-parametric Mann Whitney U performed for frontal cortical thickness in schizophrenia with methamphetamine use (SCZ_MA) and without methamphetamine use (SCZ), with *p*-values of <0.05 considered statistically significant. Effect sizes, *Biserial Correlation Coefficient ® | | | | |

Table 4: Subcortical volume group differences across schizophrenia and methamphetamine-induced psychosis using typical and atypical antipsychotics

| **Regions** | **All groups** | | | **SCZ_ATYP-**  **MAP_ATYP** | **SCZ_TYP-**  **MAP_TYP** | **SCZ_ATYP –**  **MAP_TYP** | **SCZ_TYP-MAP_ATYP** |
| --- | --- | --- | --- | --- | --- | --- | --- |
|  | *Effect size*  ${\eta\rho}^{2}$ | *CI: 90%* | *p* | *p* | *p* | *p* | *p* |
| Left hippocampus | 0.037 | (0, 0.052) | 0.84 | n.s. | n.s. | n.s. | n.s. |
| Right hippocampus | 0.064 | (0, 0.11) | 0.61 | n.s. | n.s. | n.s. | n.s. |
| Left amygdala | 0.091 | (0, 0.15) | 0.39 | n.s. | n.s. | n.s. | n.s. |
| Right amygdala | 0.074 | (0, 0.12) | 0.52 | n.s. | n.s. | n.s. | n.s. |
| Left caudate | 0.16 | (0, 0.24) | 0.10 | n.s. | n.s. | n.s. | n.s. |
| Right caudate | 0.17 | (0, 0.25) | 0.078 | n.s. | n.s. | n.s. | n.s. |
| Left putamen | 0.18 | (0, 0.27) | 0.055 | n.s. | n.s. | n.s. | n.s. |
| Right putamen | 0.16 | (0, 0.24) | 0.090 | n.s. | n.s. | n.s. | n.s. |
| Left globus pallidus | 0.11 | (0, 0.18) | 0.283 | n.s. | n.s. | n.s. | n.s. |
| Right globus pallidus | 0.17 | (0, 0.26) | 0.066 | n.s. | n.s. | n.s. | n.s. |
| Left nucleus accumbens | 0.44 | (0.21, 0.52) | < 0.0001 | 0.032 | n.s. | 0.001 | n.s. |
| Right nucleus accumbens | 0.26 | (0.048, 0.35) | < 0.0001 | n.s. | n.s. | n.s. | n.s. |
| Effect size: for all group differences. *MANOVA (F(5, 53) was performed for subcortical volumes in methamphetamine-induced psychosis on typical antipsychotics (MAP_TYP), methamphetamine-induced psychosis on atypical antipsychotics (MAP_ATYP), schizophrenia on typical antipsychotics (SCZ_TYP) and schizophrenia on atypical antipsychotics (SCZ_ATYP) groups, with *p*-values of <0.05 considered statistically significant. n.s : non-significant. Effect sizes: partial eta squared (Confidence Interval: 90%) | | | | | | | |

Table 5: Subcortical volume group differences across schizophrenia and methamphetamine-induced psychosis using typical and atypical antipsychotics versus none

| **Regions** | **All groups** | | | **SCZ_ATYP**  **SCZ_NONE** | **SCZ_TYP**  **SCZ_NONE** | **MAP_TYP-**  **MAP_NONE** | **MAP_ATY-MAP_NONE** | **SCZ_ATYP**  **MAP_NONE** | **SCZ_TYP**  **MAP_NONE** | **MAP_ATYP**  **SCZ_NONE** | **MAP_TYP**  **SCZ_NONE** | **SCZ_NONE**  **MAP_NONE** |
| --- | --- | --- | --- | --- | --- | --- | --- | --- | --- | --- | --- | --- |
|  | *Effect size*  ${\eta\rho}^{2}$ | *CI: 90%* | *p* | *p* | *p* | *p* | *p* | *p* | *p* | *p* | *p* | *p* |
| Left hippocampus | 0.037 | (0, 0.052) | 0.84 | n.s. | n.s. | n.s. | n.s. | n.s. | n.s. | n.s. | n.s. | n.s. |
| Right hippocampus | 0.064 | (0, 0.11) | 0.61 | n.s. | n.s. | n.s. | n.s. | n.s. | n.s. | n.s. | n.s. | n.s. |
| Left amygdala | 0.091 | (0, 0.15) | 0.39 | n.s. | n.s. | n.s. | n.s. | n.s. | n.s. | n.s. | n.s. | n.s. |
| Right amygdala | 0.074 | (0, 0.12) | 0.52 | n.s. | n.s. | n.s. | n.s. | n.s. | n.s. | n.s. | n.s. | n.s. |
| Left caudate | 0.16 | (0, 0.24) | 0.10 | n.s. | n.s. | n.s. | n.s. | n.s. | n.s. | n.s. | n.s. | n.s. |
| Right caudate | 0.17 | (0, 0.25) | 0.078 | n.s. | n.s. | n.s. | n.s. | n.s. | n.s. | n.s. | n.s. | n.s. |
| Left putamen | 0.18 | (0, 0.27) | 0.055 | n.s. | n.s. | n.s. | n.s. | n.s. | n.s. | n.s. | n.s. | n.s. |
| Right putamen | 0.16 | (0, 0.24) | 0.090 | n.s. | n.s. | n.s. | n.s. | n.s. | n.s. | n.s. | n.s. | n.s. |
| Left globus pallidus | 0.11 | (0, 0.18) | 0.283 | n.s. | n.s. | n.s. | n.s. | n.s. | n.s. | n.s. | n.s. | n.s. |
| Right globus pallidus | 0.17 | (0, 0.26) | 0.066 | n.s. | n.s. | n.s. | n.s. | n.s. | n.s. | n.s. | n.s. | n.s. |
| Left nucleus accumbens | 0.44 | (0.21, 0.52) | < 0.0001 | n.s. | n.s. | n.s. | n.s. | 0.0013 | n.s. | 0.046 | 0.016 | 0.0077 |
| Right nucleus accumbens | 0.26 | (0.048, 0.35) | < 0.0001 | n.s. | n.s. | n.s. | n.s. | n.s. | n.s. | n.s. | n.s. | n.s. |
| Effect size: for all group differences. MANOVA (F(5, 53) was performed for subcortical volumes in methamphetamine-induced psychosis on typical antipsychotics (MAP_TYP), methamphetamine-induced psychosis on atypical antipsychotics (MAP_ATYP), schizophrenia on typical antipsychotics (SCZ_TYP), schizophrenia on atypical antipsychotics (SCZ_ATYP), schizophrenia antipsychotic naïve (SCZ_NONE) and methamphetamine-induced psychosis antipsychotic naïve (MAP_NONE) groups, with *p*-values of <0.05 considered statistically significant. n.s : non-significant. Effect sizes: partial eta squared (Confidence Interval: 90%) | | | | | | | | | | | | |

Table 6: Frontal cortical thickness group differences across schizophrenia and methamphetamine-induced psychosis using typical and atypical antipsychotics

| **Regions** | **All groups** | | | **SCZ_ATYP-**  **MAP_ATYP** | **SCZ_TYP-**  **MAP_TYP** | **SCZ_ATYP –**  **MAP_TYP** | **SCZ_TYP-MAP_ATYP** |
| --- | --- | --- | --- | --- | --- | --- | --- |
|  | *Effect size^*#^* | *CI: 90%* | *p* | *p* | *p* | *p* | *p* |
| Left caudal middle frontal* | 0.19 | (0.0036, 0.28) | 0.04 | n.s. | n.s. | n.s. | n.s. |
| Right caudal middle frontal* | 0.12 | (0, 0.19) | 0.23 | n.s. | n.s. | n.s. | n.s. |
| Left lateral orbitofrontal^#^ | 0.61 | (0.59, 0.61) | < 0.0001 | < 0.0001 | 0.029 | n.s. | 0.0038 |
| Right lateral orbitofrontal* | 0.34 | (0.12, 0.43) | 0.0003 | 0.042 | n.s. | n.s. | n.s. |
| Left medial orbitofrontal^#^ | 0.17 | (0.16, 0.18) | 0.016 | n.s. | n.s. | n.s. | n.s. |
| Right medial orbitofrontal^#^ | 0.072 | (0.064, 0.081) | 0.12 | n.s. | n.s. | n.s. | n.s. |
| Left rostral middle frontal* | 0.15 | (0, 0.23) | 0.11 | n.s. | n.s. | n.s. | n.s. |
| Right rostral middle frontal^#^ | 0.072 | (0.17, 0.18) | 0.015 | n.s. | n.s. | n.s. | n.s. |
| Left superior frontal* | 0.18 | (0, 0.27) | 0.06 | n.s. | n.s. | n.s. | n.s. |
| Right superior frontal* | 0.21 | (0.014, 0.30) | 0.03 | n.s. | n.s. | n.s. | n.s. |
| Left frontal pole^#^ | 0.74 | (0.73, 0.75) | < 0.0001 | 0.00036 | n.s. | 0.0025 | n.s. |
| Right frontal pole^#^ | 0.092 | (0.082, 0.10) | 0.079 | n.s. | n.s. | n.s. | n.s. |
| Left pars opercularis^*^ | 0.34 | (0.12, 0,44) | 0.0003 | n.s. | n.s. | n.s. | n.s. |
| Right pars opercularis^*^ | 0.15 | (0, 0.22) | 0.13 | n.s. | n.s. | n.s. | n.s. |
| Left pars orbitalis^*^ | 0.17 | (0, 0.26) | 0.06 | n.s. | n.s. | n.s. | n.s. |
| Right pars orbitalis^*^ | 0.15 | (0, 0.23) | 0.11 | n.s. | n.s. | n.s. | n.s. |
| Left pars triangularis^*^ | 0.51 | (0.29, 0.58) | < 0.0001 | 0.0003 | n.s. | 0.034 | n.s. |
| Right pars triangularis^#^ | 0.45 | (0.44, 0.46) | < 0.0001 | 0.001 | n.s. | 0.03 | 0.015 |
| Effect size for all group differences. *MANOVA (F(5,53)) and #Kruskal Wallis ANOVA (H(5,59) performed for frontal cortical thickness in methamphetamine-induced psychosis on typical antipsychotics (MAP_TYP), methamphetamine-induced psychosis on atypical antipsychotics (MAP_ATYP), schizophrenia on typical antipsychotics (SCZ_TYP), schizophrenia on atypical antipsychotics (SCZ_ATYP), with *p*-values of <0.05 considered statistically significant. n.s : results which did not survive the MANOVA and Kruskal Wallis ANOVA. Effect sizes, *partial eta squared (Confidence Interval: 90%) and #epsilon squared (Confidence Interval: 90%) | | | | | | | |

Table 7: Frontal cortical thickness group differences across schizophrenia and methamphetamine-induced psychosis using typical, atypical and no antipsychotics

| **Regions** | **All groups** | | | **SCZ_ATYP**  **SCZ_NONE** | **SCZ_TYP**  **SCZ_NONE** | **MAP_TYP-**  **MAP_NONE** | **MAP_ATY-MAP_NONE** | **SCZ_ATYP**  **MAP_NONE** | **SCZ_TYP**  **MAP_NONE** | **MAP_ATYP**  **SCZ_NONE** | **MAP_TYP**  **SCZ_NONE** | **SCZ_NONE**  **MAP_NONE** |
| --- | --- | --- | --- | --- | --- | --- | --- | --- | --- | --- | --- | --- |
|  | *Effect size^*#^* | *CI: 90%* | *p* | *p* | *p* | *p* | *p* | *p* | *p* | *p* | *p* | *p* |
| Left caudal middle frontal* | 0.19 | (0.0036, 0.28) | 0.04 | n.s. | n.s. | n.s. | n.s. | n.s. | n.s. | n.s. | n.s. | n.s. |
| Right caudal middle frontal* | 0.12 | (0, 0.19) | 0.23 | n.s. | n.s. | n.s. | n.s. | n.s. | n.s. | n.s. | n.s. | n.s. |
| Left lateral orbitofrontal^#^ | 0.61 | (0.59, 0.61) | < 0.0001 | n.s. | n.s. | n.s. | n.s. | 0.0029 | 0.027 | n.s. | n.s. | n.s. |
| Right lateral orbitofrontal* | 0.34 | (0.12, 0.43) | 0.0003 | n.s. | n.s. | n.s. | n.s. | n.s. | n.s. | n.s. | n.s. | n.s. |
| Left medial orbitofrontal^#^ | 0.17 | (0.16, 0.18) | 0.016 | n.s. | n.s. | n.s. | n.s. | n.s. | n.s. | n.s. | n.s. | n.s. |
| Right medial orbitofrontal^#^ | 0.072 | (0.064, 0.081) | 0.12 | n.s. | n.s. | n.s. | n.s. | n.s. | n.s. | n.s. | n.s. | n.s. |
| Left rostral middle frontal* | 0.15 | (0, 0.23) | 0.11 | n.s. | n.s. | n.s. | n.s. | n.s. | n.s. | n.s. | n.s. | n.s. |
| Right rostral middle frontal^#^ | 0.072 | (0.17, 0.18) | 0.015 | n.s. | n.s. | n.s. | n.s. | n.s. | n.s. | n.s. | n.s. | 0.034 |
| Left superior frontal* | 0.18 | (0, 0.27) | 0.06 | n.s. | n.s. | n.s. | n.s. | n.s. | n.s. | n.s. | n.s. | n.s. |
| Right superior frontal* | 0.21 | (0.014, 0.30) | 0.03 | n.s. | n.s. | n.s. | n.s. | n.s. | n.s. | n.s. | n.s. | n.s. |
| Left frontal pole^#^ | 0.74 | (0.73, 0.75) | < 0.0001 | n.s. | n.s. | n.s. | n.s. | 0.00012 |  | 0.0012 | 0.0028 | 0.0004 |
| Right frontal pole^#^ | 0.092 | (0.082, 0.10) | 0.079 | n.s. | n.s. | n.s. | n.s. | n.s. | n.s. | n.s. | n.s. | n.s. |
| Left pars opercularis^*^ | 0.34 | (0.12, 0,44) | 0.0003 | n.s. | n.s. | n.s. | n.s. | n.s. | n.s. | n.s. | n.s. | n.s. |
| Right pars opercularis^*^ | 0.15 | (0, 0.22) | 0.13 | n.s. | n.s. | n.s. | n.s. | n.s. | n.s. | n.s. | n.s. | n.s. |
| Left pars orbitalis^*^ | 0.17 | (0, 0.26) | 0.06 | n.s. | n.s. | n.s. | n.s. | n.s. | n.s. | n.s. | n.s. | n.s. |
| Right pars orbitalis^*^ | 0.15 | (0, 0.23) | 0.11 | n.s. | n.s. | n.s. | n.s. | n.s. | n.s. | n.s. | n.s. | n.s. |
| Left pars triangularis^*^ | 0.51 | (0.29, 0.58) | < 0.0001 | n.s. | n.s. | n.s. | n.s. | < 0.0001 | n.s. | n.s. | n.s. | n.s. |
| Right pars triangularis^#^ | 0.45 | (0.44, 0.46) | < 0.0001 | n.s. | n.s. | n.s. | n.s. | 0.013 | 0.054 | n.s. | n.s. | n.s. |
| Effect size for all group differences. *MANOVA (F(5,53)) and #Kruskal Wallis ANOVA (H(5,59) performed for frontal cortical thickness in methamphetamine-induced psychosis on typical antipsychotics (MAP_TYP), methamphetamine-induced psychosis on atypical antipsychotics (MAP_ATYP), schizophrenia on typical antipsychotics (SCZ_TYP), schizophrenia on atypical antipsychotics (SCZ_ATYP), schizophrenia antipsychotic naïve (SCZ_NONE) and methamphetamine-induced psychosis antipsychotic naïve (MAP_NONE) groups, with *p*-values of <0.05 considered statistically significant. n.s : results which did not survive the MANOVA and Kruskal Wallis ANOVA. Effect sizes, *partial eta squared (Confidence Interval: 90%) and #epsilon squared (Confidence Interval: 90%) | | | | | | | | | | | | |
